# Stability analysis of a phase-shifted full-bridge circuit for electric vehicles based on adaptive neural fuzzy PID control

**DOI:** 10.1038/s41598-021-99559-4

**Published:** 2021-10-08

**Authors:** Yan Liu, Yan Huang, He Zhang, Qiang Huang

**Affiliations:** School of Mechanical Engineering, Northwestern Polytechnical University, Xi’an, China

**Keywords:** Energy science and technology, Engineering, Mathematics and computing

## Abstract

In the paper, adaptive neural fuzzy (ANF) PID control is applied on the stability analysis of phase-shifted full-bridge (PSFB) zero-voltage switch (ZVS) circuit, which is used in battery chargers of electric vehicles. At first, the small-signal mathematical model of the circuit is constructed. Then, by fuzzing the parameters of PID, a closed-loop system of the small-signal mathematical model is established. Further, after training samples collected from the fuzzy PID system by adaptive neural algorithm, an ANF PID controller is utilized to build a closed-loop system. Finally, the characteristics of stability, overshoot and response speed of the mathematical model and circuit model systems are analyzed. According to the simulation results of PSFB ZVS circuit, the three control strategies have certain optimizations in overshoot and adjustment time. Among them, the optimization effect of PID control in closed-loop system is the weakest. From the results of small-signal model and circuit model, the ANF PID system has highest optimization. Experiments demonstrate that the ANF PID system gives satisfactory control performance and meets the expectation of optimization design.

## Introduction

The popularization of electric vehicles is very important to solve the increasingly serious environmental problems. In practical applications, the power supply capacity and service life of electric vehicle battery pack are important for the development of electric vehicles^1^. However, the current research on battery materials has reached a bottleneck, and it is difficult to make a qualitative breakthrough, so we hope to make progress in battery charging technology^2^. In recent years, scientists have paid more attention to the charging technology, which is efficient, stable, fast, and safe to charge the power supply battery pack to improve the performance of electric vehicles in an all-round way^3^.

The commonly battery charging technology is based on controllable DC–DC conversion technology, such as Buck-Boost circuit, push–pull circuit, half-bridge DC conversion circuit, LLC circuit, isolated positive excitation circuit, etc.^4^. Forouzesh mentioned in his paper, the DC–DC conversion circuit, harmonic content, power factor, and power conversion efficiency and stability during charging are the key factors affecting the battery charging efficiency^5^. As a new technology, phase-shift full bridge (PSFB) combined with the zero-voltage switch technology (ZVS), has been proposed. The technique reduces the high frequency switch power loss and improve the power conversion efficiency greatly. In addition, closed-loop control can ensure the stability and long service life for the battery. In recent years, scientists have paid more attention to the closed-loop control strategy for PSFB ZVS DC–DC converter. The improvements of EV charger proposed by Vishnu Mahadeva Iyer, Lim and Cheon-Yong are all based on PSFB and are committed to reducing circulating current and improving the stability of the charging system^6–8^.

To ensure stability, reduce overshoot and adjustment time, an appropriate control strategy is necessary to generate PWM driver signal. PID control, as a traditional closed-loop control strategy, is used for linear systems and the results are satisfied. In a nonlinear time-varying system, it needs to be approximated linearly and difficult to get an ideal control effect. So, scientists have begun to look for more efficient methods to deal with the problems such as fuzzy PID control. Fuzzy PI control and digital fuzzy control have been proposed in mathematical model based on PSFB ZVS DC–DC conversion technology. Lo and Lin proposed a dual closed-loop voltage control for PSFB circuit^9^. Bansal and Saini applied PI control and fuzzy control separately to full bridge circuits^10^. Torok and Stig chose digital fuzzy control strategies on PSFB circuit^11^. In Bayrak’s research^12^, a robust control method was presented SMFC scheme, the control signals of the designed sliding mode fuzzy controller and the PID controller were combined which could improve loop response.

Adaptive Neural Fuzzy (ANF) inference system optimizes fuzzy control through neural network self-learning algorithm. In ANF system, the neural network self-learning algorithm could make the input and output of the fuzzy controller reach the optimal mapping relationship. The ANF control system could obtain better control effect in a short time, make up for the deficiency of fuzzy control, and make fuzzy control develop towards the direction of self-organization, self-adaptation, and self-learning^13^. So, ANF inference system has been widely used. Xiong and Shu studied the application of neural network PID control to PWM converters^14^. Chung combined ANF inference system with particle swarm optimization algorithm to control a magnetic flywheel system^15^. Kamaraj used ANF inference system to optimize brushless DC motor speed control^16^.

In the paper, fuzzy PID control technology is applied to the PSFB ZVS DC–DC converter. Then, combining back propagation algorithm and adaptive neural network control technology based on fuzzy PID control has been studied. After simulating, the stable output voltage of the circuit was obtained.

## Model of the PSFB ZVS DC–DC converter

### Electric vehicle charger structure

**Figure 1:**
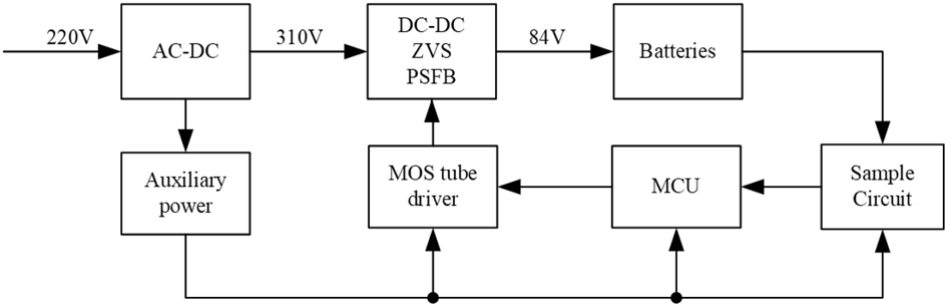
The charger structure of electric vehicle.

The rated voltage of the battery pack for an electric vehicle is 84 V when it is charging. Therefore, the charger needs to rectify 220 V AC voltage to 310 V DC voltage first, and then convert the 310 V voltage to 84 V by using the PSFB ZVS DC–DC conversion technology. The voltage conversion process is shown in Fig. 1, the system mainly consists of three parts:

1. Rectifier circuit: 220 V AC, as the input of the charger, is rectified to 310 V AC by using the bridge rectifier circuit. Then, 310 V DC is as the input voltage for the PSFB ZVS DC–DC converter.
2. PSFB ZVS DC–DC converter: 84 V DC voltage is got from the circuit. It will be used as the charge voltage for the battery pack.
3. Feedback circuit: The voltage of the battery pack is real-time monitored by MCU in system. According to the monitored data, the PWM wave generator adjusts phase-shift angles in fixed duty cycle to change the working state of MOSFET, and the output voltage of DC–DC circuit changes synchronously. DSP, such as DSPIC33FJ16GS504, is selected as the MCU of the circuit, which could complete the control methods.
4. Driver circuit: The grid-source drive voltage responsible for generating the power MOS switch tube to control the switch tube on and off.
5. Auxiliary power: Provide auxiliary power for the whole charger system.

In the structure, applying PSFB ZVS DC–DC conversion technology can improve power conversion efficiency obviously^17^.

### PSFB ZVS DC–DC converter

**Figure 2:**
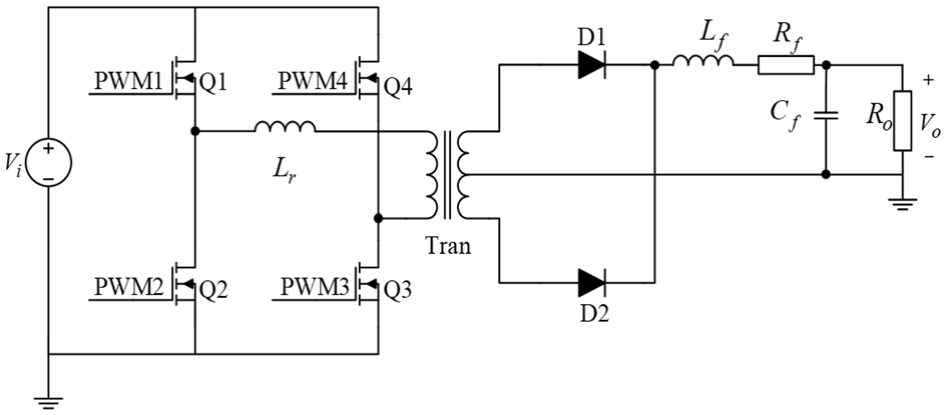
PSFB ZVS DC–DC converter.

Based on the traditional full-bridge DC–DC converter circuit, the PSFB ZVS DC–DC converter is designed. Combined with soft switching technology, the voltage falls to zero before the MOSFET is turned on or off, eliminating any overlap between voltages^18^. In this case, the defects of hard switch technique, such as energy loss and noise, are solved perfectly. As shown in Fig. 2, the opened-loop PSFB ZVS DC–DC converter consists of rectifier and inverter circuit.

Utilizing the phase-shifted control technology of PWM, the duty cycle of voltage for the original and secondary sides of the transformer are adjusted to get precise output voltage in inverter circuit. The parameters of the components in circuit are shown in Table 1.

**Table 1:** Parameters of components in circuit.

Symbol	Quantity	Value
*V*_*i*_	Input voltage	310 V
*V*_*o*_	Output voltage	84 V
*k*	Ratio	3.2
*f*_*s*_	Switch frequency	50k
*L*_*r*_	Resonant inductor	10 μH
*L*_*f*_	Filter inductor	150 μH
*R*_*f*_	Filter resistance	0.05 Ω
*C*_*f*_	Filter capacitor	3000 μF
*R*_*o*_	Load resistance	10 Ω

Simulation is carried out for the opened-loop circuit, and the simulation results are shown in Fig. 5. Deviation *E*_*e*_ and the deviation change rate *EC*_*e*_ of output voltage are used to distinguish the circuit performance. Here, *E*_*e*_(*t*) = 84 − *V*_*o*_(*t*) and *EC*_*e*_(*t*) = *de/dt*.

**Figure 3:**
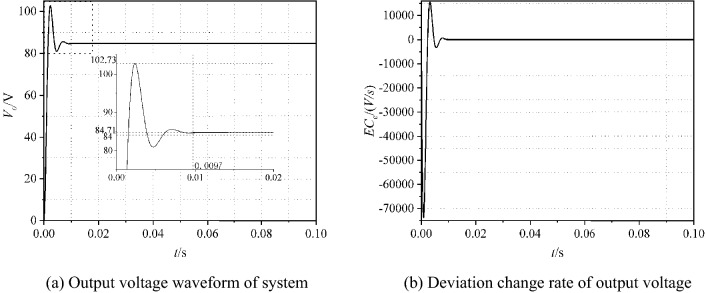
Simulation results of the opened-loop PSFD ZVS DC–DC converter.

As can be seen from Fig. 3, the output voltage fluctuates at 84 V. The maximum voltage reaches *V*_*om*_ = 102.73 V. After calculated, the overshoot is 22.3%. The time to steady state is 0.0097 s. The range of deviation change rate *EC*_*e*_ is [− 7.5 × 10^4^, 1.6 × 10^4^].

### PSFB ZVS DC–DC converter

**Figure 4:**
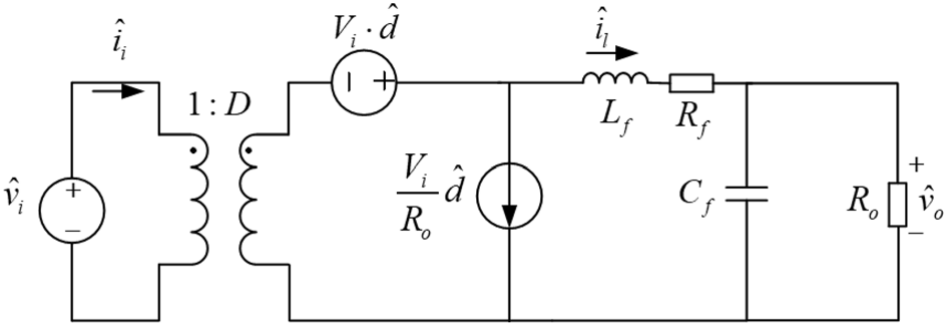
AC small-signal model of PSFB DC–DC converter.

The AC small-signal mathematical model of the circuit is constructed to design the closed-loop control system^19–22^. Figure 4 shows the AC small-signal model of PSFB DC–DC converter, which is construct by BUCK circuit based on the state space averaging method^19,23^.

Ignoring the small filter resistors *R*_*f*_, the transfer function between the duty cycle (*D*) and the output voltage (*V*_*o*_) is

1 $$ G_{vd} (s) = \frac{{\hat{v}_{o} (s)}}{{\hat{d}(s)}} = \frac{{V_{i} }}{{s^{2} L_{f} C_{f} + s\frac{{L_{f} }}{{R_{o} }} + 1}} $$

where *V*_*i*_ is the input voltage, *L*_*f*_ and *C*_*f*_ are the filter inductor and capacitor, and *R*_*o*_ is the load resistor. Considering the loss of duty cycle, the effective duty cycle of the secondary side of transformer is defined as

2 $$ D_{eff} = D - \Delta D $$

where *D* is the duty cycle of the secondary side and Δ*D* is the loss of *D*. The change of Δ*D* is related to the disturbance of the input voltage *V*_*i*_, the inductor current *I*_*l*_ and the duty cycle *D*. Let $\hat{d}_{eff}$ as the disturbance signal of *D*_*eff*_,

3 $$ \hat{d}_{eff} = \hat{d}_{i} + \hat{d}_{v} + \hat{d}_{d} $$

In Eq. (3), $\hat{d}_{i}$ is the disturbance caused by the filtered inductor current, is $\hat{d}_{v}$ the disturbance generated by the input voltage, and $\hat{d}_{d}$ is the disturbance generated by the duty cycle.

4 $$ \hat{d}_{i} = - \frac{{4 \cdot L_{r} \cdot f_{s} }}{{k \cdot V_{i} }} \cdot \hat{i}_{l} $$

5 $$ \hat{d}_{v} = \frac{{4 \cdot L_{r} \cdot I_{l} \cdot f_{s} }}{{k \cdot V_{i}^{2} }} \cdot \hat{v}_{i} $$

In Eqs. (4) and (5), *L*_*r*_ is the resonant inductance including the primary leakage inductance of the transformer, *f*_*s*_ is the switching frequency of the MOSFET, and *k* is the ratio of the transformer. Let $R_{d} = \frac{{4 \cdot L_{r} \cdot f_{s} }}{{k^{2} }}$ and substitute *R*_*d*_ to Eqs. (3)–(5), yields

6 $$ \hat{d}_{eff} = \hat{d}_{i} + \hat{d}_{v} + \hat{d}_{d} = \hat{d}_{d} - \frac{{k \cdot R_{d} }}{{V_{i} }} \cdot \hat{i}_{l} + \frac{{k \cdot R_{d} \cdot I_{l} }}{{V_{i}^{2} }} \cdot \hat{v}_{i} $$

**Figure 5:**
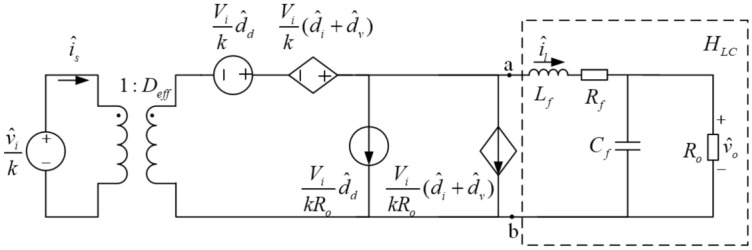
AC small-signal model of PSFB ZVS DC–DC converter.

For the AC small-signal model of PSFB DC–DC converter, there exist the loss of duty cycle occurs in the secondary side of the transformer. Therefore, the ZVS technology has been applied in the small-signal model. In in the small-signal model of PSFB ZVS DC–DC converter, *D*_*eff*_, $\hat{d}_{eff}$ and $\hat{v}_{i} /k$ are used instead of D, $\hat{d}$ and $\hat{v}_{i}$ as shown in Fig. 5.

In Fig. 5, the circuit in the virtual frame is the *LC* filter and the transfer function of the filter is

7 $$ H_{LC} (s) = \frac{{\hat{v}_{o} (s)}}{{\hat{v}_{ab} (s)}} = \frac{1}{{s^{2} L_{f} C_{f} + s\frac{{L_{f} }}{{R_{o} }} + 1}} $$

The input impedance of the *LC* filter is shown in Eq. (8),

8 $$ Z(s) = \frac{{\hat{v}_{ab} (s)}}{{\hat{i}_{l} (s)}} = \frac{{\hat{v}_{ab} (s)}}{{\hat{v}_{o} (s)}} \cdot \frac{{\hat{v}_{o} (s)}}{{\hat{i}_{l} (s)}} = \frac{{R_{o} }}{{H_{LC} (s) \cdot (sC_{f} R_{o} + 1)}} $$

The transfer function between duty cycle and output voltage is given by

9 $$ G_{vd} (s) = \frac{{\hat{v}_{o} (s)}}{{\hat{d}_{d} (s)}} = \frac{{\hat{v}_{o} (s)}}{{\hat{v}_{ab} (s)}} \cdot \frac{{\hat{v}_{ab} (s)}}{{\hat{d}_{d} (s)}} = H_{LC} (s) \cdot \frac{{\hat{v}_{ab} (s)}}{{\hat{d}_{d} (s)}} $$

Generally, the input voltage is constant in range of switching frequency bands, which means ${\hat{v}}_{i}=0$ and ${\hat{d}}_{v}=0$. According to Kirchhoff's voltage law, it can be obtained as follows,

10 $$ \hat{v}_{ab} (s) = \frac{{V_{i} \cdot \hat{d}_{d} (s) + V_{i} \cdot \hat{d}_{i} (s)}}{k} $$

Substitute Eqs. (4), (8) and *R*_*d*_ into Eq. (10), and yields,

11 $$ G_{vd} (s) = \frac{{\hat{v}_{o} (s)}}{{\hat{d}_{d} (s)}} = \frac{{\hat{v}_{o} (s)}}{{\hat{v}_{ab} (s)}} \cdot \frac{{\hat{v}_{ab} (s)}}{{\hat{d}_{d} (s)}} = H_{LC} (s) \cdot \frac{{\hat{v}_{ab} (s)}}{{\hat{d}_{d} (s)}} $$

So,

12 $$ \frac{{\hat{v}_{ab} (s)}}{{\hat{d}_{d} (s)}} = \frac{{V_{i} }}{k} \cdot \frac{Z(s)}{{R_{d} + Z(s)}} $$

Substituting Eqs. (7), (8) and (12) into (9), the transfer function of the AC small-signal model of PSFB ZVS DC–DC converter could be computed by the following formula,

13 $$ G_{vd} (s) = \frac{{V_{i} /k}}{{s^{2} L_{f} C_{f} + s\left( {\frac{{L_{f} }}{{R_{o} }} + R_{d} C_{f} } \right) + \frac{{R_{d} }}{{R_{o} }} + 1}} $$

According to the parameters of components shown in Table 1, the transfer function is calculated,

14 $$ G_{vd} (s) = \frac{96.875}{{4.5 \times 10^{ - 7} s^{2} + 6 \times 10^{ - 4} s + 1.02}} $$

**Figure 6:**
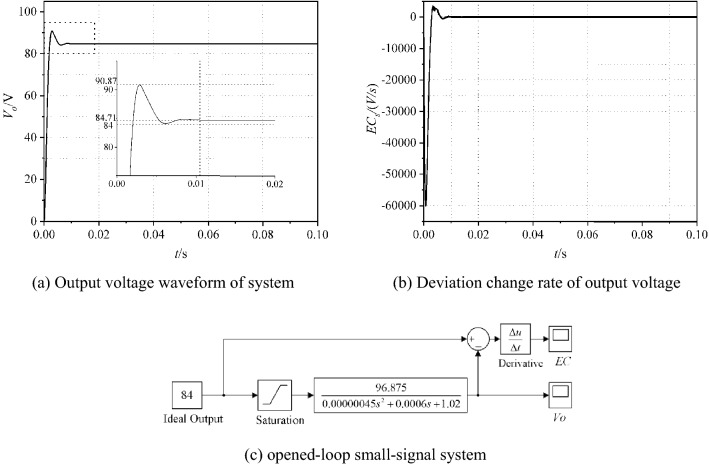
Simulation results of the opened-loop PSFD ZVS DC–DC converter.

The simulation of the opened-loop small-signal system is shown in Fig. 6c. The waveform of output voltage is like the waveform in Fig. 3. In Fig. 6, the output voltage fluctuates at 84 V. The maximum voltage reaches *V*_*om*_ = 90.87 V. After calculated, the overshoot is 8.17%. The time to steady state is 0.0105 s. The range of deviation change rate *EC*_*s*_ is [− 6.0 × 10^4^, 0.5 × 10^4^]. In addition, there exists state error in out voltage. Therefore, a closed-loop control circuit is needed to reduce overshoot and eliminate stable errors.

## Closed-loop controller design

### Parameter setting of PID controller

The key for designing the PID controller is setting *K*_*p*,_ *K*_*i*_ and *K*_*d*_. When the three parameters are set, the stability of the system should be ensured, minor overshoot and fast response time also need to be considered. The appropriate *K*_*p*_ is beneficial to reduce the error quickly while maintaining stability of the system. *K*_*i*_ can eliminate the steady-state error. Appropriate *K*_*i*_ can shorten the dynamic process of the system. *K*_*d*_ is used to predict the error tendency. Appropriate *K*_*d*_ is better for accelerating system response speed, reducing overshoot and improving stability. There are several methods for tuning a PID controller, such as manual tuning, Ziegler–Nichols method, PID tuning software packages, etc. Compared with other methods, the manual tuning, applied in this paper, is simple and easy to implement^24^.

When manually tuning is used in the PID controller, *K*_*i*_ and *K*_*d*_ values are first set to zero. After that, by increasing the value of *K*_*p*_ until the output oscillates, the value of *K*_*p*_ is 260 currently. In practice, the *K*_*p*_ should be set to approximately half of that value for a "quarter amplitude decay" type response. However, to study the optimization effect of ANF PID controller, we set *K*_*p*_ as 200. Then, the *K*_*i*_ is increased, until the steady-state error is corrected. For the system, the excessive *K*_*i*_ will cause instability and the increase of overshoot. Here, *K*_*i*_ is set as 30. Finally, *K*_*d*_ should be increased slowly to reduce the adjustment time. But *K*_*d*_ can cause oscillation. So, *K*_*d*_ = 0.01.

### Design of Sugeno fuzzy PID controller

**Figure 7:**
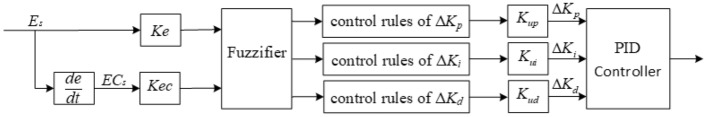
The block diagram of the fuzzy controller.

The fuzzy PID controller could optimize the PID control effect by adjusting the PID parameters in real time. The structure diagram of the fuzzy controller is shown in Fig. 7. Because ANF controller designed in the later stage requires multiple input and single output, Sugeno type is adopted. In the fuzzy PID controller, the deviation (*E*_*s*_) and the deviation change rate (*EC*_*s*_) will be input into the fuzzy controller. The map relationships between Δ*K*_*p*_, Δ*K*_*i*_, Δ*K*_*d*_ and *E*_*s*_ (/*EC*_*s*_) should be established. According to the changes of *E*_*s*_ and *EC*_*s*_, Δ*K*_*p*_, Δ*K*_*i*_, and Δ*K*_*d*_ are generated by fuzzy control rules to adjust the *K*_*p*_, *K*_*i*_ and *K*_*d*_ in PID controller.

The design of the fuzzy controller includes three steps.

1. *Fuzzification of input variables*

**Figure 8:**
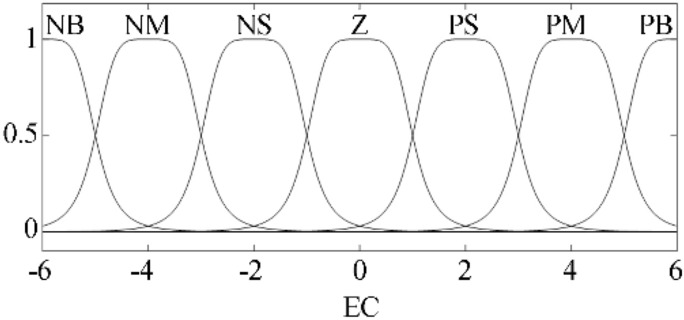
Membership function of fuzzy input variables in fuzzy PID controller.

The purpose of fuzzification is to determine the fuzzy quantization factor and membership function of input *E*_*s*_ and *EC*_*s*_. Seven fuzzy language variables (NB, NM, NS, Z, PS, PM and PB) are used to discretize *E*_*s*_ and *EC*_*s*_. According to the simulation results showed in Fig. 8, the basic domain of *E*_*s*_ is set to *X*_*e*_ $\in$[− 10, 91], and the basic domain of *EC*_*s*_ is *X*_*ec*_ $\in$[− 6.0 × 10^4^, 0.5 × 10^4^]. The fuzzy quantification domains *M* of *X*_*e*_ and *X*_*ec*_ are taken as [− 6, 6], and the quantization factors *K*_*e*_ and *K*_*ec*_ are defined in Eqs. (15) and (16).

15 $$ K_{e} = \frac{{\left| M \right|_{\max } }}{{\left| {X_{e} } \right|_{\max } }} $$

16 $$ K_{ec} = \frac{{\left| M \right|_{\max } }}{{\left| {X_{ec} } \right|_{\max } }} $$

After calculating, fuzzy quantization factors of *E*_*s*_ and *EC*_*s*_ are *K*_*e*_ = 0.059 and *K*_*ec*_ = 8.5 × 10^–5^. As shown in Fig. 8, the GBELLMF bell type membership function is used to Fuzzy the input variables. For Fuzzy PID controller, membership functions of *E*_*s*_ and *EC*_*s*_ are same.

2. *Determine the exact fuzzy output*

According the parameters set in the PID controller, we choose the scopes of Δ*K*_*p*_, Δ*K*_*i*_ and Δ*K*_*d*_ as *Y*_*p*_ $\in$[− 50, 50], *Y*_*i*_ $\in$[− 30, 30] and *Y*_*d*_ $\in$[− 0.001, 0.001] separately. Same as the fuzzy input, the seven fuzzy language variables are also used to describe Δ*K*_*p*_, Δ*K*_*i*_ and Δ*K*_*d*_. So, the corresponding values are as follows:

$$ \begin{gathered} \Delta K_{p} : \, \left\{ {{\text{NB}} = - {5}0,\;{\text{NM}} = - {33}.{4},\;{\text{NS}} = - {16}.{7},\;{\text{Z}} = 0,\;{\text{PS}} = {16}.{7},\;{\text{PM}} = {33}.{4},\;{\text{PB}} = {5}0} \right\} \hfill \\ \Delta {\text{Ki}}: \, \left\{ {{\text{NB}} = - {3}0,\;{\text{NM}} = - {2}0,\;{\text{NS}} = - {1}0,\;{\text{Z}} = 0,\;{\text{PS}} = {1}0,\;{\text{PM}} = {2}0,\;{\text{PB}} = {3}0} \right\} \hfill \\ \Delta {\text{Kd}}: \, \left\{ {{\text{NB}} = - 0.00{1},\;{\text{NM}} = - 0.000{7},\;{\text{NS}} = - 0.000{3},\;{\text{Z}} = 0,\;{\text{PS}} = 0.000{3},\;{\text{PM}} = 0.000{7},\;{\text{PB}} = 0.00{1}} \right\} \hfill \\ \end{gathered} $$

3. *Setting fuzzy control rules*

Fuzzy control rules are established based on the following principles:

When |*E*_*s*_*|* is large, to quickly reduce the deviation, it is necessary to increase *K*_*p*_ and reduce *K*_*d*_. At the same time, *K*_*i*_ is set as zero to eliminate the influence of the integral term. In this case, the large overshoot will be avoided in the regulation process. When |*E*_*s*_*|* is medium, the main task is to avoid the system shaking. So, a slightly larger *K*_*d*_ is preferable, while reducing *K*_*p*_ to avoid overshoot. When |*E*_*s*_*|* is small, it is necessary to increase *K*_*p*_ and *K*_*i*_ appropriately to reduce the adjustment time. At the same time, *K*_*d*_ should be inversely correlated with |*EC*_*s*_*|* to avoid oscillation^25–27^. According to the initial value and bisection principle, the values of Δ*K*_*p*_, Δ*K*_*i*_ and Δ*K*_*d*_ in different system states are shown in Tables 2, 3 and 4

**Table 2:** Fuzzy control rules of Δ*K*_*p*_.

*ΔK*_*p*_	*E*						
	NB	NM	NS	Z	PS	PM	PB
**EC**							
NB	− 50	− 50	− 50	− 33.4	− 33.4	0	0
NM	− 50	− 50	− 33.4	− 33.4	− 16.7	0	0
NS	− 33.4	− 33.4	− 16.7	− 16.7	0	16.7	16.7
Z	− 33.4	− 16.7	− 16.7	0	16.7	16.7	33.4
PS	− 16.7	− 16.7	0	16.7	16.7	33.4	33.4
PM	0	0	33.4	33.4	33.4	50	50
PB	0	0	16.7	33.4	50	50	50

**Table 3:** Fuzzy control rules of Δ*K*_*i*_.

*ΔK*_*i*_	*E*						
	NB	NM	NS	Z	PS	PM	PB
**EC**							
NB	30	30	20	20	10	0	0
NM	30	20	20	20	10	0	0
NS	20	20	20	10	0	− 10	− 20
Z	20	10	10	0	− 10	− 20	− 20
PS	10	10	0	− 10	− 10	− 20	− 20
PM	0	0	− 10	− 20	− 20	− 20	− 30
PB	0	− 10	− 10	− 20	− 20	− 30	− 30

**Table 4:** Fuzzy control rules of Δ*K*_*d*_.

*ΔK*_*d*_		E						
		NB	NM	NS	Z	PS	PM	PB
EC	NB	a	a	b	b	b	d	d
	NM	− a	− a	− a	− a	b	− a	d
	NS	− d	− d	− c	− a	b	a	c
	Z	− d	− c	− c	− a	b	a	c
	PS	− c	− c	− a	− a	b	a	a
	PM	− c	− a	− a	− a	b	a	a
	PB	a	b	b	b	b	d	d

a = 0.0003; b = 0; c = 0.0007; d = 0.001.

**Figure 9:**
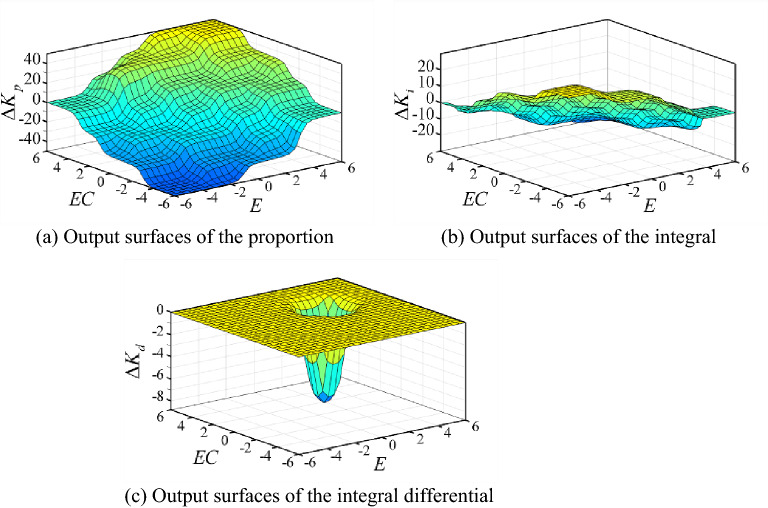
Output surfaces of the coefficients for fuzzy PID controller.

According to the above principles, the fuzzy control rules are obtained. The output surfaces of the proportion, the integral and differential coefficients on the domain, respectively, are shown in Fig. 9.

Due the fuzzy control rules of fuzzy PID are fixed, the fuzzy PID control strategy cannot adjust the initial PID parameters accurately, so it is not satisfied the expectation of the system. Therefore, an ANF controller has been applied to optimize the closed-loop system.

### Design of Sugeno adaptive neural fuzzy PID controller

For ANF PID controller, the key is training fuzzy control rules by sample data from fuzzy control system. According to the simulation result shown in Figs. 14 and 16, the fuzzy PID control has a better stability control effect for small-signal and circuit model. So, the simulation data from fuzzy PID controller can be used as sample data. There are 1000 data sets as sample data to train the Sugeno fuzzy controller generated in the progress of designing of Sugeno Fuzyy PID controller. As one of neural network self-learning algorithm, back propagation algorithm is utilized to generate ANF PID controller.

**Figure 10:**
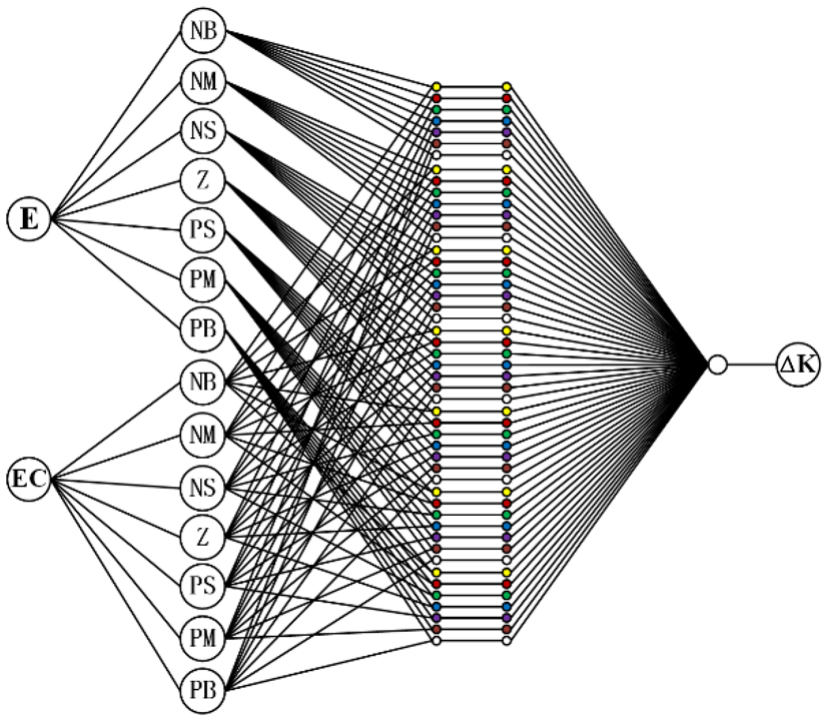
Adaptive neural fuzzy control network.

There are five layers of the ANF control network shown in Fig. 10. The meanings and functions of each layer are as follows:

1. The first layer is the input layer which is composed of two neural nodes. The layer is responsible for transmitting *E*_*s*_ and *EC*_*s*_ to the next layer.
2. The second layer, composed of two groups of neural nodes, describes the membership function of the fuzzy input. The neural nodes represent the fuzzy language variables of *E*_*s*_ and *EC*_*s*_ respectively. This layer is responsible for obscuring the input.
3. The third layer is the ANF control rule layer, which is composed of 49 neural nodes. Each node represents a fuzzy rule.
4. The fourth layer is the membership function of the fuzzy output. The layer is also composed of 49 neural nodes corresponding to the 49 control rules in the third layer. In the layer, each node represents the weight of the corresponding rules.
5. The fifth layer is the output layer. In the layer, each control rule is linearly combined to obtain the fuzzy output. Backpropa algorithm was used to optimize the weight in fourth layer. To achieve the expected result, the error tolerance value is set as 0.1, and the upper limit of iteration number is set as 10,000. After 10,000 iterations, the error is reduced to 0.074616, which could meet the training purpose.

**Figure 11:**
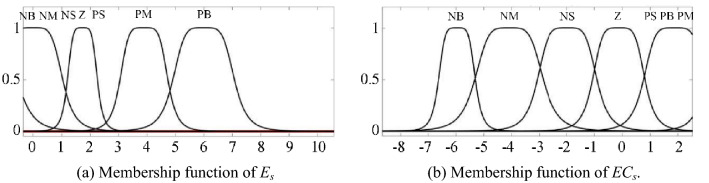
Membership function of fuzzy input variables in ANF PID controller.

Membership functions of fuzzy input variables in ANF PID controller are shown in Fig. 11. Compare with Fig. 8, shapes of membership function are different between fuzzy PID controller and ANF PID controller. For *E*_*s*_ and *EC*_*s*_, because of the difference in the weight, the shapes of membership functions are also different in adaptive neural fuzzy PID controller.

**Figure 12:**
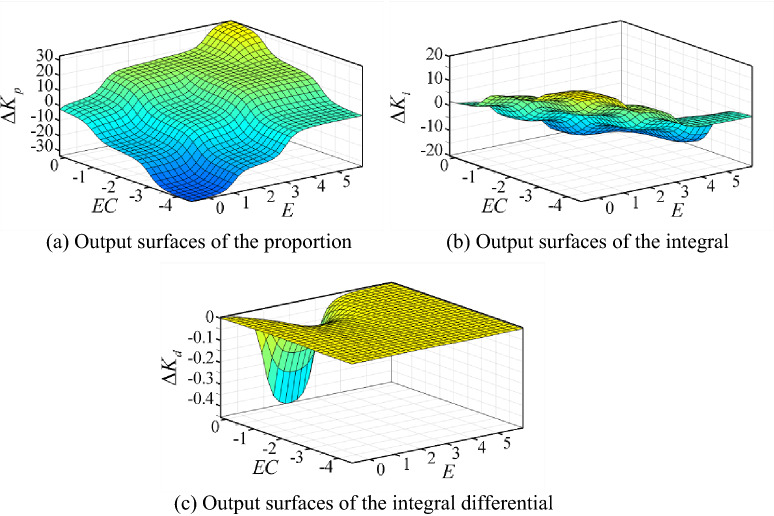
Output surfaces of the coefficients for adaptive neural fuzzy PID controller.

For adaptive neural fuzzy PID controller, the output surfaces of the proportion, the integral and differential coefficients on the domain, respectively, are shown in Fig. 12. The fuzzy control rules adjust timely based on the real states of system. So, the control effect could meet the expectation of the system.

In a circuit model of adaptive neural fuzzy PID control, the control system can real-time actual output voltage circuit and the ideal output voltage deviation between *E* and *EC* input deviation rate adaptive neural fuzzy controller, the adaptive neural fuzzy controller based on fuzzy control rules of Δ*K*_*p*_, Δ*K*_*i*_, Δ*K*_*d*_, real-time adjustment of PID control parameters, the optimized PID controller to the control effect of the circuit.

After self-learning training, the control rules will be applied to PSFB ZVS DC–DC model to simulate the optimization effect.

## Simulation and analysis of the closed-loop system

### Closed-loop control for the small-signal model

**Figure 13:**
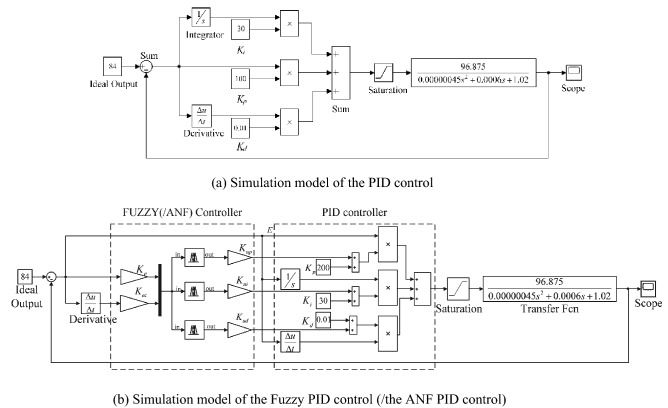
Simulation model of closed-loop system.

PID control is used in the AC small-signal model of the PSFB ZVS DC–DC circuit shown in Fig. 13a, and Fuzzy (/ANF) PID control system is shown in Fig. 13b. Except for the different fuzzy control rules, the structure of ANF PID system and fuzzy PID system are same. In Fig. 13b, according to the error of *V*_*o*_, the Fuzzy (/ANF) controller adjusts the parameters of PID to modify the input of the small-signal model. Under these strategies, the response of closed-loop system could be optimized.

**Figure 14:**
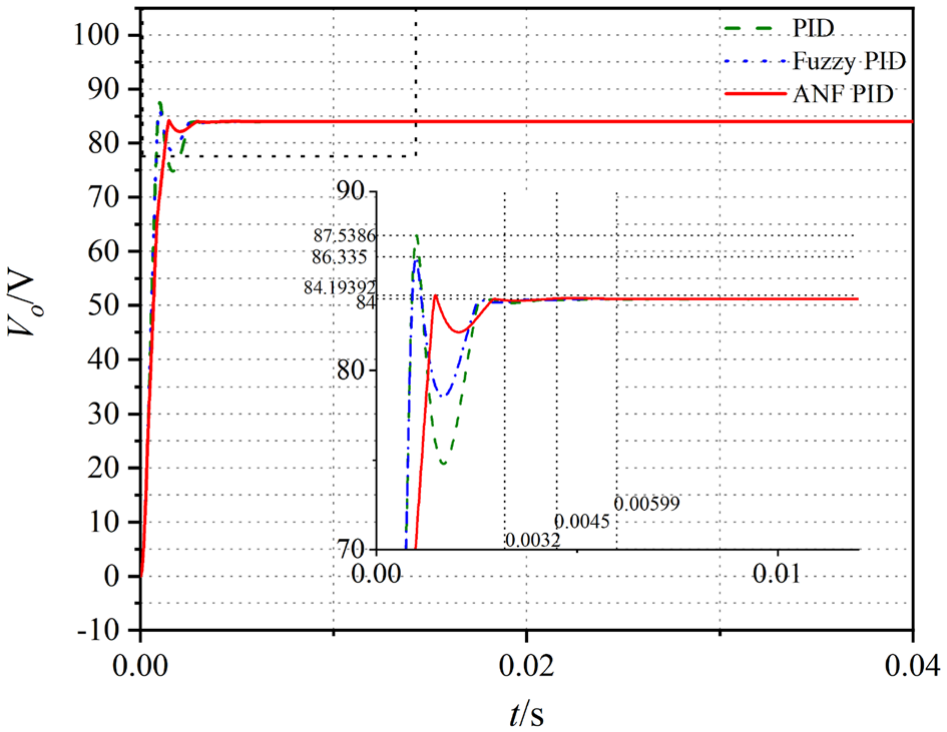
Voltage waveform in closed loop small-signal model.

The output voltages of the systems for the small-signal model are shown in Fig. 14. Compared with Fig. 6, the small overshoot and short adjustment time could be gotten in closed-loop control system. For the three control strategies, the optimization effect of PID control is the weakest.

The performances of the opened-loop and closed-loop control systems with different methods are shown in Table 5. To compare the effectivity of the methods, the optimization rate of overshoot *G* is defined as

17 $$ G = \frac{{\sigma_{o} - \sigma_{c} }}{{\sigma_{o} }}\% $$

**Table 5:** Evaluation of small signal model.

Method	*t*_*s*_/s	*t*_*r*_/s	*σ*%	*G*
Opened-loop	0.0105	0.0009	8.2%	–
PID	0.00599	0.0009	4.2%	48%
Fuzzy PID	0.0045	0.0014	2.8%	66%
ANF PID	0.0032	0.0021	0.3%	96%

*t*_*s*_ time to steady state, *t*_*r*_ rise time, *σ%* overshoot amount, *G* optimization rata of overshoot.

In Eq. (17), ${\sigma }_{o}$ means the overshoot of the opened loop system and ${\sigma }_{c}$ is the overshoot of the closed loop system with different PID controllers.

According to the response curves and characteristic values shown in Fig. 14 and Table 5, the time to steady state, over-shoot and optimization rate of the output voltage are greatly improved when closed-loop controller is applied. Compared with the other two controllers, ANF PID controller has highest optimization rate which could reach to 96%. The time to steady state for ANF PID controller is only 0.0032 s which is much faster than that in opened-loop system.

From simulation results of the small-signal model, the ANF PID controller is more adaptive to optimize the control system than the two other methods. It could be expected that the ANF PID control strategy is effective for the PSFB ZVS DC–DC closed-loop circuit.

**Figure 15:**
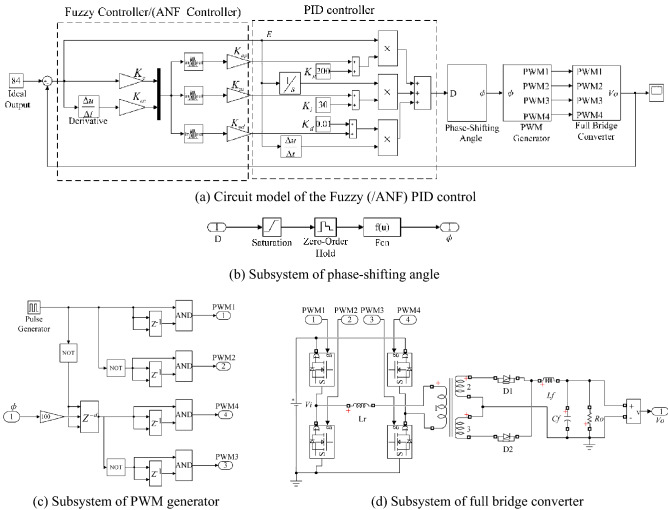
Circuit model of the closed-loop system.

**Figure 16:**
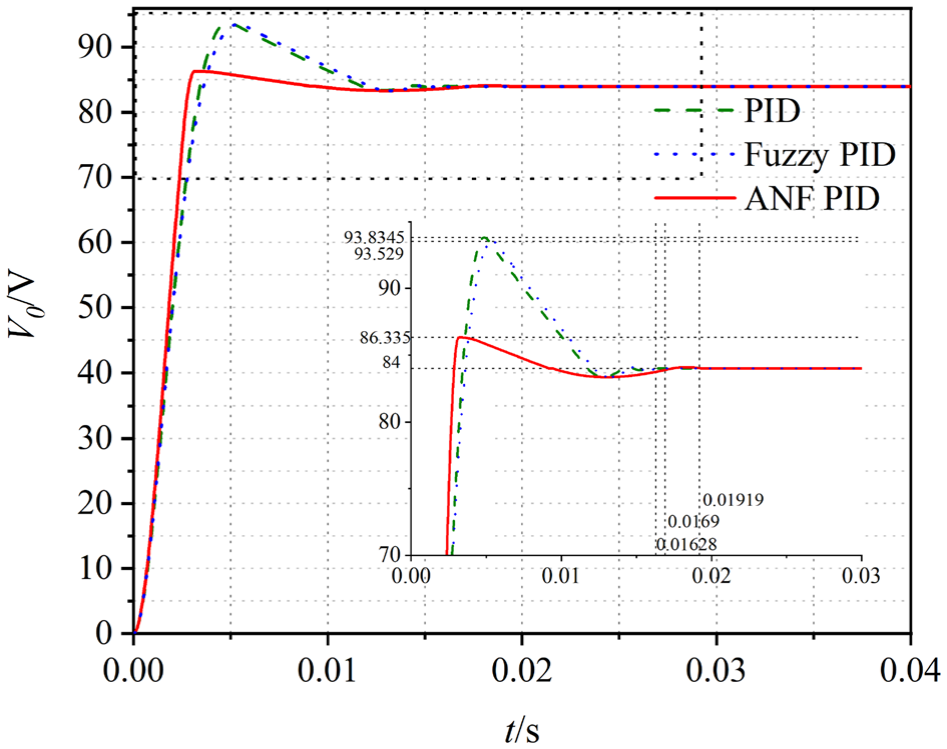
Voltage waveform in closed loop small-signal model.

The simulation model of the circuit system is shown in Fig. 15. The control performances of the circuit models are shown in Table 6. Similar with small-signal model, the over-shoot and optimization rate of the output voltage are greatly improved when closed-loop controller is applied in circuit model. In these three closed-loop systems, ANF PID system has highest optimization rate which could reach to 87.4%. Compared with PID, fuzzy PID has little improvement in overshoot optimization rate. After the adaptive optimization, the overshoot suppression effect of ANF PID system has been improved. For circuit model, the time to steady state for ANF PID controller is 0.0192 s which is slightly longer than that in the other systems. This result is different from that of the small-signal model. The may reason for the differences is that the circuit model is a kind of physical model in which the components exist inherent response time. The excessive overshoot will seriously affect charging system of electric vehicles performance and shorten its life. Therefore, it can be inferred that the ANF PID control system meet the requirements of PSFB ZVS DC–DC circuit (Fig. 16).

**Table 6:** Evaluation of small signal model.

Method	*t*_*s*_/s	*t*_*r*_/s	*σ*%	*G*
Opened-loop	0.0097	0.0015	22.3%	–
PID	0.0163	0.0034	11.7%	48.9%
Fuzzy PID	0.0169	0.0026	11.3%	49.3%
ANF PID	0.0192	0.0028	2.8%	87.4%

## Conclusion

The simulations of the small-signal model and circuit model for PSFB ZVS DC–DC circuit are analyzed using the PID, fuzzy PID and ANF PID control. The ANF PID control method proposed in this paper can dynamically adjust the PID parameters in the system transition process to obtain better control effect. The stabilities, responding speed and overshoot of the system are regulated by adjusting the factors of the control strategy. The ANF PID control method can restrain the overshoot to 2.8%, and the adjusting time is increased compared with the PID and Fuzzy PID control methods, but it is still within the expected range and the small overshoot can prolong the battery life. From the results of the simulations for small-signal model and circuit model, the effect of ANF PID control in the three closed-loop control system is optimal.

In a conclusion, the ANF PID controller can be applied to the electric vehicle battery charging technology, which will improve the anti-interference performance of the circuit and enhance the stability of the charging process. But adaptive neural fuzzy controller adopted in this paper needs more complicated data training and the hardware of this control strategy is difficult to be realized. These are our next research.

Fortunately, dynamic adjustment of circuit parameters through machine learning method to achieve better control effect should be a more meaningful direction in the development of control. In the future, the methods and results of simulation may be used to the practical circuits to design an efficient and stable electric vehicle battery charger.
